# Supplementary material for: Quantification of Nervonic Acid in Human Milk in the First 30 Days of Lactation: Influence of Lactation Stages and Comparison with Infant Formulae
Source: Nutrients. 2019 Aug 14;11(8):1892. doi: 10.3390/nu11081892 (PMC6723218; doi:10.3390/nu11081892)
Supplement: Supplementary file 1 [file nutrients-11-01892-s001.pdf]

**Table S1.** Nervonic acid (mg/g fat) concentration in human milk during the first month of lactation.

| Lactation (days) | Sample 1 | Sample 2 | Sample 3 | Sample 4 | Sample 6 | Sample 7 | Sample 8 | Range | Average | *p* value value |
|---|---|---|---|---|---|---|---|---|---|---|
| 3 | 0.77 ± 0.02$^{a}$ | 1.21 ± 0.02$^{a}$ | 1.23 ± 0.19$^{a}$ | 0.72 ± 0.02$^{a}$ | 1.44 ± 0.02$^{a}$ | 0.86 ± 0.06$^{a}$ | 0.87 ± 0.06$^{a}$ | 0.72-1.44 | 1.00 ± 0.24$^{a}$ | *** |
| 4 | 0.67 ± 0.01$^{b}$ | 1.01 ± 0.04$^{b}$ | 1.00 ± 0.04$^{b}$ | 0.67 ± 0.01$^{b}$ | 1.04 ± 0.00$^{b}$ | 0.74 ± 0.05$^{b}$ | 0.63 ± 0.02$^{b}$ | 0.63-1.04 | 0.80 ± 0.17$^{b}$ | ** |
| 5 | 0.59 ± 0.01$^{c}$ | 0.83 ± 0.01$^{c}$ | 0.86 ± 0.02$^{c}$ | 0.57 ± 0.01$^{c}$ | 0.81 ± 0.09$^{c}$ | 0.65 ± 0.00$^{c}$ | 0.54 ± 0.03$^{c}$ | 0.54-0.87 | 0.67 ± 0.13$^{c}$ | *** |
| 6 | 0.52 ± 0.03$^{cd}$ | 0.74 ± 0.02$^{d}$ | 0.73 ± 0.01$^{d}$ | 0.53 ± 0.00$^{d}$ | 0.59 ± 0.09$^{d}$ | 0.58 ± 0.01$^{d}$ | 0.48 ± 0.04$^{d}$ | 0.44-0.74 | 0.57 ± 0.10$^{d}$ | *** |
| 7 | 0.47 ± 0.01$^{de}$ | 0.62 ± 0.02$^{e}$ | 0.66 ± 0.00$^{de}$ | 0.51 ± 0.00$^{d}$ | 0.47 ± 0.04$^{e}$ | 0.51 ± 0.02$^{e}$ | 0.41 ± 0.02$^{e}$ | 0.37-0.66 | 0.50 ± 0.09$^{e}$ | *** |
| 8 | 0.44 ± 0.01$^{de}$ | 0.57 ± 0.01$^{e}$ | 0.56 ± 0.03$^{ef}$ | 0.47 ± 0.02$^{e}$ | 0.34 ± 0.00$^{f}$ | 0.47 ± 0.00$^{e}$ | 0.38 ± 0.04$^{e}$ | 0.34-0.57 | 0.45 ± 0.08$^{f}$ | *** |
| 9 | 0.42 ± 0.02$^{e}$ | 0.50 ± 0.03$^{f}$ | 0.44 ± 0.03$^{f}$ | 0.42 ± 0.03$^{f}$ | 0.28 ± 0.01$^{fg}$ | 0.45 ± 0.03$^{e}$ | 0.37 ± 0.03$^{e}$ | 0.28-0.50 | 0.40 ± 0.06$^{g}$ | *** |
| 10 | 0.39 ± 0.02$^{e}$ | 0.37 ± 0.01$^{g}$ | 0.29 ± 0.03$^{g}$ | 0.41 ± 0.02$^{f}$ | 0.23 ± 0.00$^{gh}$ | 0.44 ± 0.02$^{e}$ | 0.28 ± 0.00$^{f}$ | 0.23-0.45 | 0.35 ± 0.07$^{h}$ | *** |
| 11-15 | 0.26 ± 0.04$^{f}$ | 0.34 ± 0.02$^{g}$ | 0.24 ± 0.01$^{g}$ | 0.25 ± 0.03$^{g}$ | 0.20 ± 0.01$^{ghi}$ | 0.30 ± 0.04$^{f}$ | 0.27 ± 0.02$^{f}$ | 0.20-0.34 | 0.27 ± 0.04$^{i}$ | * |
| 16-20 | 0.25 ± 0.09$^{f}$ | 0.23 ± 0.02$^{h}$ | 0.21 ± 0.04$^{g}$ | 0.23 ± 0.00$^{gh}$ | 0.17 ± 0.01$^{hi}$ | 0.21 ± 0.01$^{g}$ | 0.18 ± 0.01$^{g}$ | 0.17-0.27 | 0.22 ± 0.03$^{j}$ | NS |
| 21-25 | 0.21 ± 0.05$^{f}$ | 0.20 ± 0.02$^{h}$ | 0.19 ± 0.02$^{g}$ | 0.21 ± 0.03$^{hi}$ | 0.13 ± 0.02$^{hi}$ | 0.20 ± 0.01$^{g}$ | 0.17 ± 0.00$^{g}$ | 0.13-0.24 | 0.20 ± 0.04$^{jk}$ | NS |
| 26-30 | 0.20 ± 0.04$^{f}$ | 0.21 ± 0.06$^{h}$ | 0.15 ± 0.03$^{g}$ | 0.19 ± 0.03$^{i}$ | 0.16 ± 0.01$^{i}$ | 0.17 ± 0.02$^{g}$ | 0.15 ± 0.01$^{g}$ | 0.14-0.22 | 0.18 ± 0.03$^{k}$ | NS |

Different superscript lowercase letters indicate significant differences ($p < 0.05$) with a column. *p* values indicate differences between eight mothers: ***, $p < 0.001$; **, $p<0.01$; *, $p < 0.05$; NS, $p > 0.05$.

**Table S2.** Total fat content (mg/mL) in human milk during the first month of lactation.

| Lactation (days) | Sample 1 | Sample 2 | Sample 3 | Sample 4 | Sample 6 | Sample 7 | Sample 8 |
|---|---|---|---|---|---|---|---|
| 3 | 16.00 ± 1.22 | 10.65 ± 0.36 | 17.69 ± 0.19 | 13.75 ± 0.78 | 10.00 ± 0.36 | 14.33 ± 1.00 | 9.50 ± 0.46 |
| 4 | 20.75 ± 1.63 | 12.83 ± 1.00 | 21.50 ± 0.69 | 17.00 ± 1.11 | 16.75 ± 1.00 | 19.00 ± 0.23 | 11.50 ± 1.23 |
| 5 | 22.50 ± 0.95 | 17.51 ± 1.45 | 22.75 ± 0.23 | 18.25 ± 0.26 | 18.00 ± 1.56 | 21.50 ± 1.04 | 15.00 ± 1.03 |
| 6 | 24.00 ± 1.32 | 22.64 ± 1.37 | 23.50 ± 0.39 | 19.25 ± 0.39 | 22.75 ±1.23 | 23.00 ± 0.99 | 19.25 ± 1.45 |
| 7 | 24.50 ± 0.65 | 23.05 ± 0.33 | 24.75 ± 0.57 | 20.50 ± 1.03 | 23.00 ± 0.56 | 27.00 ± 2.11 | 21.50 ± 0.46 |
| 8 | 26.00 ± 1.11 | 24.49 ± 0.16 | 26.50 ± 1.00 | 21.50 ± 0.26 | 24.00 ± 1.11 | 28.50 ± 0.65 | 22.50 ± 0.66 |
| 9 | 27.00 ± 0.22 | 26.52 ± 0.32 | 28.75 ±1.26 | 24.00 ± 1.33 | 25.00 ± 0.26 | 30.00 ± 1.06 | 24.25 ± 0.33 |
| 10 | 28.00 ± 0.36 | 27.41 ± 0.13 | 30.25 ± 1.49 | 25.25 ± 0.59 | 26.75 ± 1.03 | 30.67 ± 0.22 | 28.00 ± 1.26 |
| 11-15 | 30.68 ± 1.01 | 28.48 ± 0.19 | 32.05 ± 1.28 | 27.50 ± 1.22 | 28.40 ± 0.96 | 34.63 ± 1.11 | 32.87 ± 1.59 |
| 16-20 | 35.50 ± 1.36 | 32.32 ± 1.22 | 35.90 ± 2.16 | 33.80 ± 1.56 | 34.65 ± 2.36 | 42.95 ± 2.26 | 37.68 ± 2.05 |
| 21-25 | 39.44 ± 2.03 | 37.84 ± 2.03 | 38.35 ± 1.69 | 37.98 ± 1.04 | 37.56 ± 1.33 | 45.35 ± 1.26 | 39.86 ± 1.11 |
| 26-30 | 40.65 ± 2.33 | 39.66 ± 1.03 | 40.19 ± 1.22 | 39.89 ± 2.10 | 40.33 ± 2.04 | 49.57 ± 1.11 | 40.26 ± 0.22 |

**Table S3.** Total fatty acid composition (%, wt) in human milk during the lactation days.

| Fatty acids | 3 d (n = 9) | 4 d (n = 9) | 5 d (n = 9) | 6 d (n = 9) | 7 d (n = 9) | 8 d (n = 10) | 9 d (n = 7) | 10 d (n = 9) | 11-15 d (n = 43) | 16-20 d (n = 41) | 21-25 d (n = 45) | 26-30 d (n = 40) |
|---|---|---|---|---|---|---|---|---|---|---|---|---|
| 10:0 | 0.56 ± 0.26 | 0.69 ± 0.25 | 0.88 ± 0.23 | 1.08 ± 0.24 | 1.16 ± 0.22 | 1.10 ± 0.28 | 1.36 ± 0.25 | 1.30 ± 0.25 | 1.22 ± 0.17 | 1.17 ± 0.21 | 1.06 ± 0.22 | 1.03 ± 0.23 |
| 12:0 | 3.07 ± 1.14 | 3.92 ± 1.14 | 4.80 ± 1.24 | 5.79 ± 1.05 | 5.89 ± 1.15 | 5.78 ± 1.35 | 5.99 ± 1.51 | 5.74 ± 1.23 | 5.20 ± 1.24 | 4.94 ± 1.00 | 4.29 ± 1.13 | 4.17 ± 1.02 |
| 13:0 | 0.05 ± 0.02 | 0.05 ± 0.02 | 0.04 ± 0.02 | 0.05 ± 0.02 | 0.04 ± 0.02 | 0.05 ± 0.03 | 0.04 ± 0.00 | 0.05 ± 0.02 | 0.05 ± 0.02 | 0.06 ± 0.03 | 0.08 ± 0.02 | 0.04 ± 0.01 |
| 14:0 | 4.08 ± 1.37 | 4.72 ± 1.20 | 5.44 ± 1.65 | 6.20 ± 1.36 | 6.13 ± 1.70 | 5.98 ± 1.74 | 5.53 ± 1.36 | 5.27 ± 1.12 | 5.62 ± 0.90 | 4.53 ± 0.76 | 4.03 ± 0.11 | 4.21 ± 0.22 |
| 15:0 | 0.13 ± 0.02 | 0.13 ± 0.03 | 0.13 ± 0.04 | 0.14 ± 0.04 | 0.14 ± 0.03 | 0.14 ± 0.02 | 0.11 ± 0.02 | 0.12 ± 0.02 | 0.19 ± 0.12 | 0.13 ± 0.02 | 0.13 ± 0.03 | 0.14 ± 0.03 |
| 16:0 | 21.27 ± 1.36 | 20.39 ± 1.81 | 20.23 ± 1.35 | 19.90 ± 1.71 | 19.81 ± 1.12 | 20.26 ± 1.33 | 20.23 ± 2.02 | 20.02 ± 1.19 | 19.71 ± 1.68 | 21.18 ± 1.64 | 20.74 ± 2.19 | 20.64 ± 1.72 |
| 17:0 | 0.25 ± 0.03 | 0.26 ± 0.03 | 0.26 ± 0.03 | 0.26 ± 0.03 | 0.25 ± 0.05 | 0.26 ± 0.04 | 0.22 ± 0.03 | 0.25 ± 0.03 | 0.24 ± 0.03 | 0.25 ± .03 | 0.25 ± 0.04 | 0.25 ± 0.05 |
| 18:0 | 5.14 ± 0.96 | 4.98 ± 0.96 | 5.30 ± 1.13 | 5.16 ± 1.25 | 5.07 ± 1.16 | 5.40 ± 1.19 | 5.23 ± 1.41 | 5.27 ± 1.13 | 5.38 ± 0.98 | 5.39 ± 1.04 | 5.65 ± 0.92 | 5.21 ± 1.42 |
| 20:0 | 0.15 ± 0.08 | 0.17 ± 0.11 | 0.15 ± 0.12 | 0.19 ± 0.10 | 0.16 ± 0.10 | 0.17 ± 0.11 | 0.19 ± 0.11 | 0.21 ± 0.10 | 0.20 ± 0.13 | 0.21 ± 0.12 | 0.18 ± 0.11 | 0.27 ± 0.13 |
| 22:0 | 0.03 ± 0.01 | 0.03 ± 0.01 | 0.04 ± 0.02 | 0.04 ± 0.02 | 0.03 ± 0.01 | 0.08 ± 0.04 | 0.03 ± 0.01 | 0.04 ± 0.02 | 0.04 ± 0.02 | 0.04 ± .02 | 0.04 ± 0.02 | 0.05 ± 0.03 |
| 24:0 | 0.39 ± 0.19 | 0.34 ± 0.10 | 0.26 ± 0.07 | 0.23 ± 0.06 | 0.21 ± 0.05 | 0.21 ± 0.04 | 0.17 ± 0.04 | 0.17 ± 0.03 | 0.16 ± 0.03 | 0.15 ± 0.03 | 0.15 ± 0.03 | 0.14 ± 0.02 |
| 14:1 n-5 | 0.05 ± 0.01 | 0.04 ± 0.02 | 0.05 ± 0.02 | 0.14 ± 0.23 | 0.06 ± 0.02 | 0.06 ± 0.01 | 0.05 ± 0.02 | 0.05 ± 0.02 | 0.07 ± 0.02 | 0.06 ± 0.03 | 0.06 ± 0.02 | 0.06 ± 0.01 |
| 16:1 n-7 | 1.96 ± 0.57 | 1.98 ± 0.60 | 1.95 ± 0.61 | 1.96 ± 0.66 | 2.06 ± 0.53 | 2.16 ± 0.53 | 2.07 ± 0.47 | 2.02 ± 0.49 | 2.04 ± 0.36 | 2.17 ± 0.37 | 2.24 ± 0.34 | 2.47 ± 0.34 |
| 18:1 n-9 | 33.86 ± 2.59 | 32.55 ± 1.70 | 31.55 ± 2.11 | 30.23 ± 2.24 | 31.04 ± 1.50 | 30.71 ± 2.02 | 31.04 ± 1.93 | 30.99 ± 1.55 | 32.10 ± 2.13 | 31.75 ± 1.99 | 33.23 ± 2.68 | 32.66 ± 2.60 |
| 20:1 n-9 | 0.17 ± 0.01 | 0.16 ± 0.04 | 0.14 ± 0.04 | 0.13 ± 0.04 | 0.12 ± 0.03 | 0.12 ± 0.03 | 0.10 ± 0.02 | 0.09 ± 0.03 | 0.09 ± 0.03 | 0.08 ± 0.04 | 0.06 ± 0.04 | 0.06 ± 0.05 |
| 22:1 n-9 | 0.19 ± 0.06 | 0.18 ± 0.06 | 0.17 ± 0.04 | 0.17 ± 0.06 | 0.16 ± 0.06 | 0.16 ± 0.03 | 0.13 ± 0.05 | 0.13 ± 0.05 | 0.13 ± 0.05 | 0.13 ± 0.0 | 0.12 ± 0.05 | 0.11 ± 0.04 |
| 24:1 n-9 | 0.21 ± 0.07 | 0.21 ± 0.05 | 0.20 ± 0.04 | 0.18 ± 0.02 | 0.17 ± 0.03 | 0.16 ± 0.03 | 0.14 ± 0.04 | 0.13 ± 0.02 | 0.12 ± 0.02 | 0.10 ± 0.02 | 0.06 ± 0.01 | 0.04 ± 0.01 |
| 18:2 n-6 | 22.02 ± 1.78 | 22.45 ± 2.22 | 22.75 ± 2.75 | 22.98 ± 2.11 | 23.13 ± 2.03 | 23.16 ± 2.47 | 23.24 ± 2.74 | 23.25 ± 2.97 | 23.50 ± 2.31 | 23.55 ± 3.53 | 23.87 ± 3.71 | 23.99 ± 2.91 |
| 18:3 n-6 | 0.68 ± 0.19 | 0.65 ± 0.15 | 0.56 ± 0.08 | 0.52 ± 0.06 | 0.51 ± 0.06 | 0.53 ± 0.07 | 0.46 ± 0.07 | 0.46 ± 0.06 | 0.48 ± 0.05 | 0.50 ± 0.05 | 0.51 ± 0.07 | 0.53 ± 0.08 |
| 20:2 n-6 | 1.03 ± 0.31 | 0.97 ± 0.13 | 0.85 ± 0.08 | 0.78 ± 0.10 | 0.75 ± 0.09 | 0.72 ± 0.08 | 0.67 ± 0.09 | 0.64 ± 0.06 | 0.64 ± 0.09 | 0.60 ± 0.07 | 0.56 ± 0.08 | 0.55 ± 0.07 |
| 20:4 n-6 | 0.92 ± 0.13 | 0.89 ± 0.11 | 0.80 ± 0.14 | 0.78 ± 0.14 | 0.76 ± 0.11 | 0.75 ± 0.12 | 0.72 ± 0.10 | 0.70 ± 0.08 | 0.68 ± 0.10 | 0.65 ± 0.10 | 0.60 ± 0.07 | 0.62 ± 0.07 |

| | | | | | | | | | | | | |
|---|---|---|---|---|---|---|---|---|---|---|---|---|
| 18:3 n-3 | 1.41 ± 0.37 | 1.45 ± 0.34 | 1.46 ± 0.32 | 1.60 ± 0.37 | 1.62 ± 0.21 | 1.66 ± 0.26 | 1.73 ± 0.33 | 1.80 ± 0.37 | 1.86 ± 0.26 | 1.89 ± 0.27 | 1.94 ± 0.34 | 1.99 ± 0.31 |
| 20:3 n-3 | 0.62 ± 0.16 | 0.64 ± 0.16 | 0.62 ± 0.20 | 0.61 ± 0.15 | 0.63 ± 0.19 | 0.59 ± 0.17 | 0.56 ± 0.16 | 0.60 ± 0.21 | 0.58 ± 0.19 | 0.57 ± 0.19 | 0.49 ± 0.13 | 0.47 ± 0.13 |
| 20:5 n-3 | 0.11 ± 0.02 | 0.10 ± 0.02 | 0.09 ± 0.02 | 0.08 ± 0.02 | 0.09 ± 0.01 | 0.08 ± 0.02 | 0.07 ± 0.01 | 0.14 ± 0.06 | 0.07 ± 0.01 | 0.07 ± 0.02 | 0.08 ± 0.02 | 0.08 ± 0.02 |
| 22:6 n-3 | 0.72 ± 0.30 | 0.71 ± 0.19 | 0.66 ± 0.12 | 0.64 ± 0.14 | 0.62 ± 0.16 | 0.61 ± 0.15 | 0.60 ± 0.18 | 0.60 ± 0.18 | 0.57 ± 0.16 | 0.56 ± 0.11 | 0.50 ± 0.18 | 0.42 ± 0.23 |
| SFAs | 35.11 ± 5.45 | 35.69 ± 5.66 | 37.52 ± 5.90 | 39.03 ± 5.88 | 38.90 ± 5.62 | 39.42 ± 6.27 | 39.10 ± 6.90 | 38.44 ± 5.13 | 38.51 ± 3.16 | 38.04 ± 1.28 | 36.60 ± 2.13 | 37.44 ± 1.58 |
| MUFAs | 36.34 ± 3.40 | 35.04 ± 2.47 | 33.96 ± 2.86 | 32.74 ± 3.25 | 33.55 ± 2.17 | 33.31 ± 2.65 | 33.50 ± 2.53 | 33.40 ± 2.15 | 34.54 ± 2.59 | 34.31 ± 3.22 | 35.85 ± 1.44 | 34.52 ± 2.13 |
| PUFAs | 28.66 ± 4.30 | 29.28 ± 4.80 | 28.52 ± 4.54 | 28.18 ± 3.50 | 27.56 ± 3.54 | 27.31 ± 3.24 | 27.44 ± 2.97 | 28.18 ± 3.41 | 26.56 ± 1.57 | 27.58 ± 2.55 | 27.52 ± 1.49 | 28.16 ± 2.14 |
| LA/ALA | 16.53 ± 8.10 | 16.51 ± 9.94 | 16.16 ± 9.04 | 14.46 ± 6.67 | 16.07 ± 3.25 | 15.37 ± 8.39 | 16.07 ± 6.45 | 15.69 ± 6.20 | 15.09 ± 2.45 | 15.48 ± 1.53 | 15.11 ± 2.53 | 16.15 ± 1.59 |
| n-6/n-3 | 9.32 ± 5.28 | 9.09 ± 5.81 | 9.31 ± 5.37 | 8.54 ± 3.86 | 8.89 ± 5.73 | 8.58 ± 3.30 | 9.44 ± 3.90 | 8.90 ± 2.79 | 8.72 ± 3.02 | 9.18 ± 2.16 | 9.57 ± 1.49 | 10.34 ± 2.13 |
